# Guinea worm eradication: Progress and challenges— should we beware of the dog?

**DOI:** 10.1371/journal.pntd.0005495

**Published:** 2017-04-20

**Authors:** David Molyneux, Dieudonné P. Sankara

**Affiliations:** 1 Department of Parasitology, Liverpool School of Tropical Medicine, Pembroke Place, Liverpool, United Kingdom; 2 Department of Control of Neglected Tropical Diseases, World Health Organization, Geneva, Switzerland; University of Washington, UNITED STATES

The Global Guinea Worm Eradication (Dracunculiasis) Programme has made spectacular progress since it began in the 1980’s. The numbers of individuals afflicted by Guinea Worm disease has declined from an estimated 3.5 million cases in 1986 to only 25 cases in 2016 [1,2]. The disease is caused by infection with *Dracunculus medinensis* and acquired by ingestion of infected water fleas, *Cyclops* and *Mesocyclops* species. The objective of the programme is the Global Eradication of the infection; WHO defines eradication as **“the permanent reduction to zero of the worldwide incidence of infection caused by a specific pathogen as a result of deliberate efforts with no risk of reintroduction”.** It is important to emphasise the total absence of global transmission of the infection and that no specific host is specified in the definition [3]. The programme was initiated in 1980 just before the International Water and Sanitation Decade (1981–1990) recognizing that although rare animal infections had been recorded only human hosts of *D*. *medinensis* were considered significant [4]. Animal infections have disappeared following cessation of human transmission [5]. The eradication of the infection was thus considered technically feasible using public health measures, despite the absence of any suitable medical intervention, as it was considered there was no animal reservoir. Because Guinea worm disease was not well known as a condition there was a need for advocacy to communicate the severe impact of the infection on the poorest individuals and remotest communities. Since 1986, the engagement of former U.S. President Jimmy Carter and the involvement of the Carter Center provided significant momentum for the eradication programme. The Carter Center, working with WHO, UNICEF and the United States Centers for Disease Control and Prevention (CDC) has been a critical leadership, advocacy and fund raising force which brought Guinea worm disease and the eradication programme to the attention of policy makers and eradication is now within our grasp [5,6].

The eradication programme has been based on simple principles-provision of improved water sources, use of water filtration using different types of cloth or filament filters, health education to inform populations of how the infection is acquired and can be prevented, control of the intermediate host copepod using the larvicide, Abate, (temephos), containment of cases before they have an opportunity to contaminate water sources, active surveillance in endemic or previously endemic villages, provision of rewards to increase awareness and encourage reporting and the rapid (within 24 hours) follow up of any rumours and a robust and regular reporting system [6,7].

Twenty-one countries were endemic in the 1980s. (Until South Sudan gained its independence on 9 July 2011, it was part of Sudan; thus, between the 1980s and 2011, 20 countries were endemic for the disease.) The global incidence in these countries was estimated to be 3.5 million in 1986 [1,6]. However, in 1989, almost 900,000 cases were detected by country surveys in villages in endemic countries.

Progress towards eradication requires countries to request WHO to undertake Certification Team missions to assess the absence of transmission. Assessment is based on a detailed country report and extensive examination of the data presented, together with detailed scrutiny and field evaluations of the country’s programme over the duration of the implementation of the programme.

At the end of 2016, 17 of the 21 previously endemic countries had stopped transmission of the disease, out of which 15 have been certified free of transmission by the WHO. This was further to successful certification missions led by members of WHO-appointed International Commission for the Certification of Dracunculiasis Eradication (ICCDE) [3] along with recommendation by the ICCDE to the Director-general that such countries had achieved transmission free status. In fact, every country must submit to WHO a statement that they are free of Guinea Worm transmission; to date 186 countries and territories have been certified as free of Guinea Worm transmission by WHO upon ICCDE’s recommendations.

Currently four endemic countries remain–Chad, Ethiopia, Mali and South Sudan which reported a total of 25 human cases in 2016 [2]. However, Mali did not report any human infection in 2016, for the first time ever since it began its eradication programme in 1991. Kenya and Sudan, which were previously endemic and have not reported a case for at least three years, are preparing to request certification of being free of Guinea worm disease; an International Certification Team will visit these countries to verify if they have fulfilled the requisite steps to be certified as transmission free. Angola and the Democratic Republic of Congo (DRC), countries which have had no history of Guinea worm disease since the 1980s, also require to be assessed as to whether these countries are free from transmission, before each of these countries submit a dossier for certification [3].

Despite this impressive progress, major challenges to eradication- “the permanent reduction to zero of the worldwide incidence of *D*. *medinensis* infection” have emerged. Chad, which, over a period of 10 years between 2000 and 2010, did not report a single human case, started to report cases which did not reflect the existing knowledge of the pattern of transmission; despite these cases not being contained they did not appear to give rise to other cases, 10–14 months following the emergence of a worm. A similar pattern continued for two years before it was recognized that dogs were infected, as demonstrated by emerging Guinea worms [8]. Molecular identification of dogs' worms showed they were indistinguishable from the worms found in human cases in Chad [8,9]. It rapidly became clear that many dogs were infected as the numbers of detected infections rose significantly between 2014 and 2016. 503 infected dogs were found in 2015 while in 2016 1011 dogs have been reported as being infected [2]. The focus of the canine outbreak in Chad is along the Chari River (see map in [2]). The hypothesis proposed for the peculiar epidemiology is that due to the ecology of the area and behaviour of the local human population, who exploit the abundance of fish through seasonal fish harvesting, that an alternative life cycle pertains in Chad where *D*. *medinensis* uses a paratenic or transport host. *Dracunculus* species that infect wild carnivores (racoons and otters) in the USA and Canada have a paratenic fish host, hence the possibility that a similar cycle exists in Chad. Initially, this was believed to be in fish, which would become infected by consuming the infected copepods and then harbour viable infective larvae [8]. Experimental evidence has since shown that amphibians can also harbour viable *D*. *medinensis* larvae [10] but recently an infected frog has been found in Chad supporting the hypothesis that paratenic hosts contribute to *Dracunculus* epidemiology in that setting [11]. A suggested approach to control transmission was to educate the population as to how to dispose of fish entrails (to prevent dogs acquiring infection as they forage) and to ensure the fish were dried and cooked effectively to kill infective *D*.*medinensis* larvae. The situation in Chad appears to be unique and various measures to address the situation have been initiated. Rewards for reporting dog infections and tethering infected dogs to prevent their access to water sources, burying fish entrails, using the drug, Heartgard (ivermectin used to control canine heartworm, *Dirofilaria immitis*, in the USA) as a potential chemoprophylactic drug to prevent infection and vigorous use of Abate (temephos) at watering points on the edges of the lagoons which are considered the source of infected *Cyclops*. The emergence of so many dog infections in Chad is a setback not only for Chad but poses a risk to neighbouring countries previously declared free of transmission, in particular Central African Republic, but also Cameroon, Niger and Nigeria, reflecting the need to maintain surveillance in all countries previously certified as free of transmission until Global Eradication is declared.

Of further concern is that a small number of infected dogs have been identified in Ethiopia (as well as baboons in Ethiopia) and Mali and appear to act as susceptible hosts whether infection is acquired via a paratenic host, as suggested in Chad or by the classical direct acquisition of infection by ingesting water containing infected *Cyclops*. While the ecology in affected areas of Mali (inland Niger delta) is similar to areas along the Chari River in Chad, the ecology in the affected area of Ethiopia appears different from Chad and calls into question whether a paratenic host is relevant there. Whatever the local life cycle, the common feature is an obligatory requirement for an intermediate copepod host. This should be the focus of interventions-aggressive efforts to reduce *Cyclops* populations in all water bodies, notwithstanding the need to deploy all the other interventions that have been so successful to date. In addition we need to identify the role of dogs as “reservoirs” of infection. Can dogs act as a sustained source of human infections?

The major objective over the coming years is to certify the global cessation of transmission of *D*. *medinensis*. This will require certification in 8 remaining countries each in a different status. Evidence that there is a complete absence of transmission not only of the parasite to humans but to other susceptible animal hosts will be required. Proving the absence of transmission in the light of the different transmission patterns together with the security concerns are a challenge. Staff of International Organizations and NGOs are often unable to travel to verify the reports which are required for certification due to insecurity. However, Ministries of Health with the support of WHO and the Carter Center, must focus on interrupting transmission by the vigorous pursuit of copepod control, the containment of human cases and dog infections and through the application of what we know works. Whilst the infections in dogs are a setback it is essential that if permanent reduction to zero of the worldwide incidence is to be achieved that the end game is effectively financed and appropriate field research is undertaken to understand the different emerging local epidemiology.
